# Biomechanical Analysis of Angled Abutments in the Anterior Maxilla: Impact of Bone Quality and Loading Conditions Using Finite Element Analysis

**DOI:** 10.7759/cureus.84329

**Published:** 2025-05-18

**Authors:** Jaspinder Kaur, Sandeep Kumar, Rajnish Aggarwal, Ashutosh R Singh, Mansi Sharma, Rahul Sharma, Pamei Jenthuilu, Kulashekar Reddy Nandalur

**Affiliations:** 1 Department of Prosthodontics, Surendera Dental College and Research Institute, Sri Ganganagar, IND; 2 Department of Oral and Maxillofacial Surgery, Dr. S.S. Tantia Medical College, Hospital and Research Centre, Sri Ganganagar, IND; 3 Prosthetic Dental Sciences, College of Dentistry, Jazan University, Jazan, SAU

**Keywords:** angled, anterior, finite element analysis, implants, maxilla, stresses

## Abstract

Objective: This in vitro study utilized three-dimensional (3D) finite element analysis (FEA) to investigate the stress distribution around dental implants in the anterior maxilla, focusing on the interplay between 15*°* angled abutments, bone quality (D1-D4), and loading conditions (axial and oblique).

Methodology: Anatomically accurate 3D models of the anterior maxilla, a titanium Internal-Hex implant system (Touareg^TM^(S), Adin Dental Implants, Chile), a 15*°* angled abutment, and a porcelain-fused-to-metal crown were developed using cone-beam computed tomographic (CBCT) scans and CATIA V5 software (Dassault Systèms, Vélizy-Villacoublay, France) and then analyzed in ANSYS Workbench (ANSYS Inc., Canonsburg, Pennsylvania, USA). Four bone types (D1-D4) were modeled with varying cortical and cancellous proportions. Axial (178 N) and oblique (100 N at 120*°*) loads were applied to eight models, and the stress and strain distributions were evaluated at the implant-bone and implant-abutment interfaces using tetrahedral elements. Mesh convergence ensured computational accuracy, and the material properties reflected clinical data.

Results: Axial loading showed uniform stress at the implant neck, with cortical bone stress increasing from 12.023 MPa (D1) to 26.438 MPa (D4) and implant stress increasing from 67.319 MPa to 72.291 MPa. Oblique loading amplified stress asymmetrically, with cortical bone stress increasing from 20.005 to 30.704 MPa and implant stress from 97.910 to 102.458 MPa. D3 and D4 exhibit significantly higher stresses, particularly under oblique loading.

Conclusion: Bone quality and loading type critically influenced stress distribution. The stresses were higher in the D3 and D4 bones under oblique loading. Bone D1 and D2 provided robust support, whereas bones D3 and D4 required enhanced implant designs or augmentation. The 15*°* angled abutment increased stress, necessitating careful prosthetic planning to ensure biomechanical stability and esthetic outcomes in anterior maxillary implant dentistry.

## Introduction

Dental implants have transformed anterior tooth restorations in which esthetics and function are paramount. Bone loss after tooth extraction often reduces bone volume and density in the anterior maxilla, challenging implant stability and long-term success [1]. Clinicians must choose between bone augmentation and strategic implant placement in areas with better bone quality [2]. Implant-supported restorations are favored because of their durability and ability to restore both function and appearance in the anterior region. However, anatomical constraints such as the nasal cavity in the anterior maxilla [3] frequently require angled abutments to achieve optimal prosthetic alignment [4].

Angled abutments address non-parallel implant placement, enabling functional and esthetically pleasing restorations. Krekmanov et al. [5] studied 47 patients and reported a 98% success rate with tilted maxillary implants after five years compared to 93% for non-tilted implants. However, angled abutments introduce biomechanical challenges, as deviation from the implant’s long axis increases stress at the implant-abutment interface and crestal bone [6]. Pre-angled abutments (15°-35°) meet prosthetic demands but may concentrate stress, potentially leading to bone loss or compromised osseointegration [6,7].

Bone quality is a key determinant of implant success in the anterior region, where bone density varies. Misch and Zudy [8] classified bone by density, i.e., D1 and D2 types, which are common in the anterior mandible, provide strong stability, while D3 and D4 types, prevalent in the anterior maxilla, are less dense and offer weaker support. These differences affect stress distribution and implant integration, particularly in the anterior region, where esthetic precision is critical [9]. The elastic modulus of the cortical bone helps manage stress at the crestal interface, and excessive stress can trigger bone resorption or implant failure. Low-density bone in the anterior maxilla increases the risk of stress-related complications [10].

Finite element analysis (FEA) has been widely used to study the stress distribution around implants; however, most studies have focused on standard implant positions [11,12]. Meijer et al. [11] and Papavasiliou et al. [12] analyzed the stress around implants under axial loading and reported increased stress concentrations in softer bone types. While these studies provide valuable insights, they often overlook the combined effects of angled abutments and varying bone quality in the anterior jaws [11]. 

This in vitro study used three-dimensional (3D) FEA to investigate the stress distribution around dental implants in the anterior maxilla. It focuses on the interplay of angled abutments, bone quality (D1 to D4), and loading conditions (axial and oblique). Unlike previous studies, which primarily examined standard implant placements or posterior regions, this study targeted the anterior region, where esthetic and biomechanical demands are unique. This study aimed to evaluate the influence of angled abutments and varying bone densities on stress patterns under axial and oblique loading, providing insights into optimizing implant placement, abutment selection, and treatment planning for enhanced outcomes in anterior implant dentistry.

This in vitro study modeled the interactions among a titanium implant, a 15° angled abutment, and surrounding bone of varying qualities (D1, D2, D3, and D4), as classified by Misch and Judy [8]. This study aimed to provide insights into implant stability, stress behavior, and longevity by simulating clinical scenarios with different bone densities and loading conditions (axial and oblique), thereby guiding clinical decisions regarding implant placement and abutment selection in the anterior maxilla. 

## Materials and methods

Development of the FEA model

Using cone-beam computed tomography (CBCT), anatomically accurate 3D digital models of the anterior maxillary region were created using the CATIA V5 software (Dassault Systèms, Vélizy-Villacoublay, France). As CBCT scans were obtained from the database, institutional ethical committee clearance was waived for this study, and written informed consent was obtained from the patient to use their records for study purposes, maintaining confidentiality. The anterior maxillary bone was modeled to reflect the four bone types (D1-D4), representing the range of densities encountered in clinical practice. The D1 bone, characterized by a dense cortical structure, offers superior mechanical support, while the D2 bone, which is common in the anterior maxilla, features dense trabecular bone with a thick cortical layer [10]. The D3 bone consists of moderate-density cortical and trabecular mix, and D4, often seen in older patients, is a soft trabecular bone with minimal cortical thickness [8]. CBCT scans provide a detailed 3D geometry of the anterior maxilla, capturing bone heterogeneity. The bone block was designed with dimensions of 14 mm (base to crestal bone), 8 mm mesiodistally, and 8 mm buccolingually, based on anatomical norms. Cortical thicknesses were derived from Katranji, Misch, and Wang [13], reporting buccal and palatal thicknesses of 1.04 ± 0.29 mm and 1.36 ± 1.45 mm, respectively, in the edentulous maxilla. Bone type proportions were set as D1 (75%), D2 (60%), D3 (45%), and D4 (25%), with D1 (6 mm cortical, 2 mm cancellous; buccal 2.8 mm, palatal 3.2 mm), D2 (4.8 mm cortical, 3.2 mm cancellous; buccal 2.06 mm, palatal 2.74 mm), D3 (3.6 mm cortical, 4.4 mm cancellous; buccal 1.5 mm, palatal 2.6 mm), and D4 (2 mm cortical, 6 mm cancellous; buccal 0.8 mm, palatal 1.4 mm) [13].

Implant and abutment design

A titanium dental implant was digitally modelled using specifications derived from the Adin Internal-Hex implant system (TouaregTM(S), Adin Dental Implants, Chile), which is known for its superior thread design and mechanical performance. The implant measured 11.5 mm in length with a diameter of 4.2 mm. The implant featured a collar height of 1.5 mm, thread pitch of 1.2 mm, thread height of 0.7 mm, major diameter of 4.2 mm, and tip diameter of 2.0 mm, designed to optimize osseointegration. A 7 mm high titanium abutment with an angulation of 15° was attached to simulate clinical scenarios in which anatomical or esthetic constraints necessitated tilted placement [14]. A 15° angulation of a premanufactured abutment has the potential to establish parallelism between neighboring abutments [14]. The abutment, which was connected to a porcelain-fused-to-metal (PFM) crown, transmitted forces to the implant. In order to replicate the prosthetic apparatus, a PFM crown was fabricated for the left maxillary central incisor, measuring 10.5 mm in height and 8.5 mm in buccolingual width, and a corresponding model of this crown was likewise produced.

Creation of FEA models

**Figure 1:**
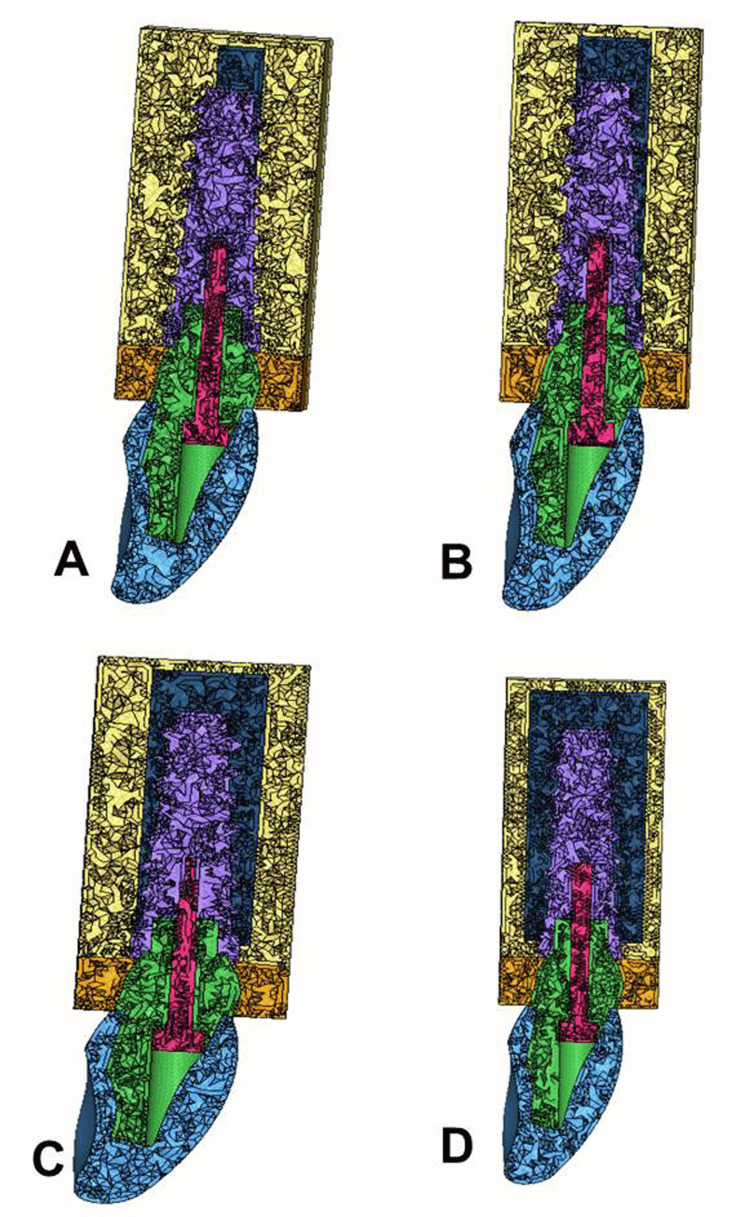
Finite element analysis (FEA) models of maxillary anterior region: A) angled abutment in the D1 bone, B) angled abutment in the D2 bone, C) angled abutment in the D3 bone, D) angled abutment in the D4 bone. This figure illustrates the finite element analysis (FEA) models developed in the study.

3D models of the bone, implant, abutment, and crown were created and imported into ANSYS Workbench (ANSYS Inc., Canonsburg, Pennsylvania, USA), a software specialized in advanced FEA. The models were discretized into fine tetrahedral elements to ensure computational accuracy. The mesh was refined at critical interfaces (implant-bone and implant-abutment) to capture detailed stress and strain distributions, whereas larger elements were used in less critical areas to optimize the computational efficiency. Eight models were created, representing combinations of four bone types (D1, D2, D3, and D4) (Figure 1) and two loading conditions (axial and oblique), to comprehensively assess biomechanical interactions.

Mesh convergence analysis

A mesh convergence study was conducted to ensure the accuracy of FEA results. The mesh density was incrementally increased starting with a coarse mesh (approximately 50,000 elements) and progressing to finer meshes (up to 200,000 elements). Convergence was achieved when the difference in the maximum von Mises stress between consecutive mesh refinements was less than 5%, indicating sufficient accuracy [15]. The final mesh consisted of approximately 150,000 tetrahedral elements. The meshing element type was a tetrahedron with ten nodal elements.

Material properties

The material properties were defined based on established biomechanical data [16,17]. Cortical and cancellous bone parameters were derived from the literature, with adjustments made according to bone quality [16]. All materials, including the cortical bone, trabecular bone, titanium implant, and titanium abutment, were believed to be isotropic, homogeneous, elastic, and linear. The material properties are listed in Table 1.

**Table 1:** Material properties used in the finite element analysis (FEA). GPa: gigapascals., Ni-Cr: nickel-chromium for porcelain-fused-to-metal crown (PFM)

Structure	Modulus of elasticity (GPa)	Poisson’s ratio
Cancellous bone [16]	9.5 for D1, 5.5 for D2, 1.6 for D3, 0.69 for D4	0.30 for all bone types
Cortical bone [16]	14.8	0.30
Mucosa [16]	10	0.40
Titanium abutment and implant [16]	110	0.35
Ni-Cr [17]	203.6	0.30

Loading and boundary conditions

Two loading conditions were applied to simulate functional forces in the anterior maxilla. For axial loading, a 178 N vertical force was applied along the implant’s long axis at the abutment, mimicking straight-down chewing forces typical of incisor function. For oblique loading, a force of 100 N was applied at an angle of 120° to the long axis of the implant on the palatal aspect of the maxillary incisor, representing dynamic forces during angled chewing or speech. These magnitudes were based on previously reported masticatory forces in the anterior region [18]. The loads were transmitted through the PFM crown to the abutment to ensure realistic force distribution.

Precise boundary conditions were established to ensure a realistic simulation of the clinical scenarios. The base of the bone block was fully constrained in all directions (X, Y, and Z axes) to simulate the fixation of the maxillary bone within the skull, preventing any displacement during the application of masticatory forces. This rigid fixation mimics the anatomical environment in which the surrounding craniofacial structures restrict the motion of the maxillary segment. All components of the system, implant, abutment, bone, and prosthesis, were modelled under the assumption of perfect bonding, especially at the implant-bone interface, representing complete osseointegration [19]. This idealized condition eliminated the possibility of micromovement or interfacial slip and allowed an accurate interpretation of how forces are transmitted through the structure. No frictional or contact gaps were modelled at any interface to simplify the computational process and focus solely on the biomechanical behavior of the implant system.

Numerical analysis

Using ANSYS, FEA was performed to evaluate the von Mises stress distribution and strain patterns. Stress maps were used to identify high-stress regions in the implant, abutment, abutment screw, and bone, particularly at the crestal bone and implant-bone interface, to predict failure sites. Strain distributions provided insights into material deformation and implant-bone interactions. The models were assessed for excessive stress or deformation, which could indicate material failure or implant instability.

## Results

**Figure 2:**
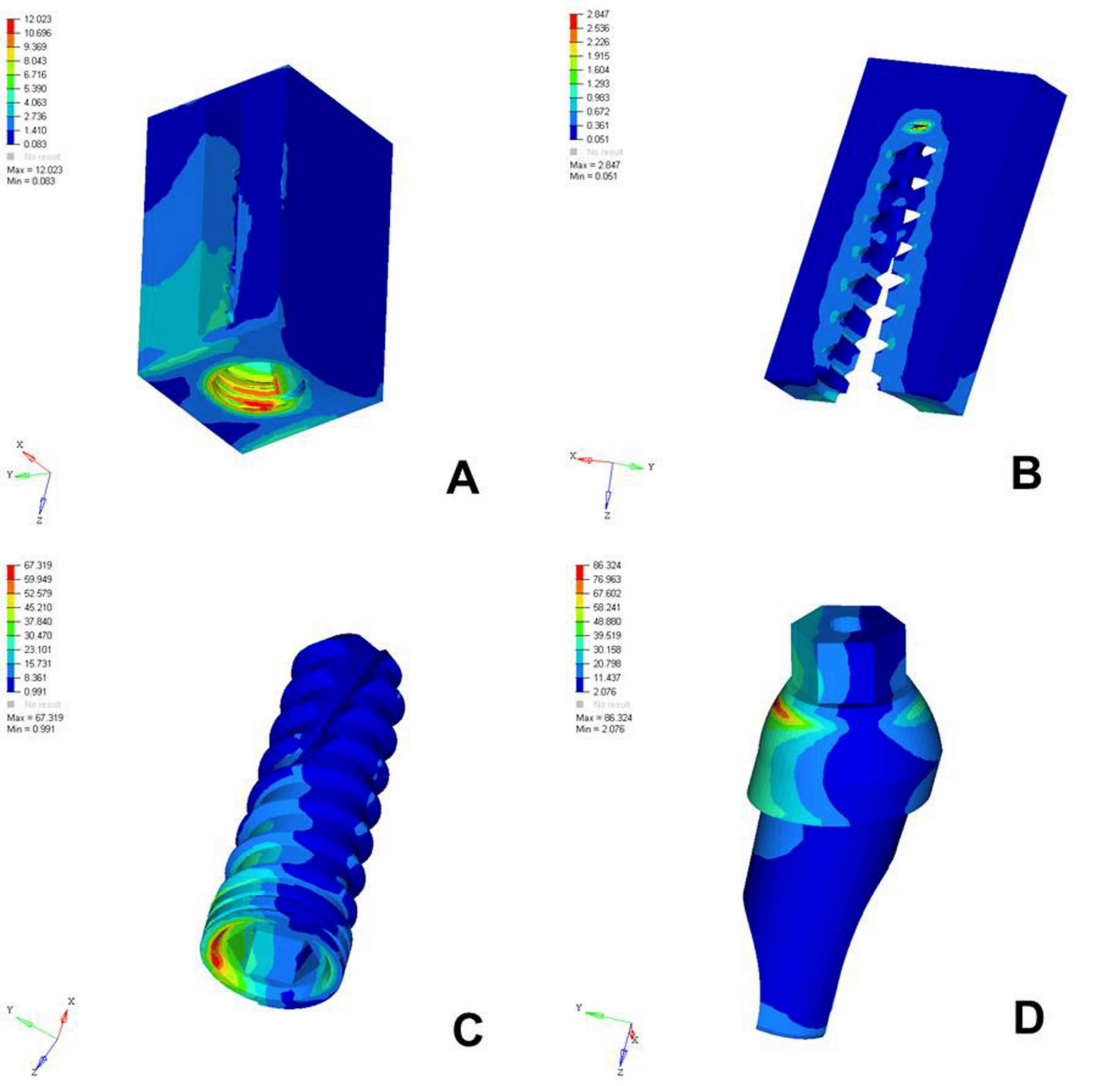
von Mises stresses in megapascals (MPa) in the D1 bone under axial loading (175 N) on various structures: A) cortical bone, B) cancellous bone, C) implant, D) abutment. The side bar shows von Mises stress from minimum (blue) to maximum (red) value in MPa. This figure illustrates the finite element analysis (FEA) model developed in the study, taken directly from the ANSYS workbench.

**Figure 3:**
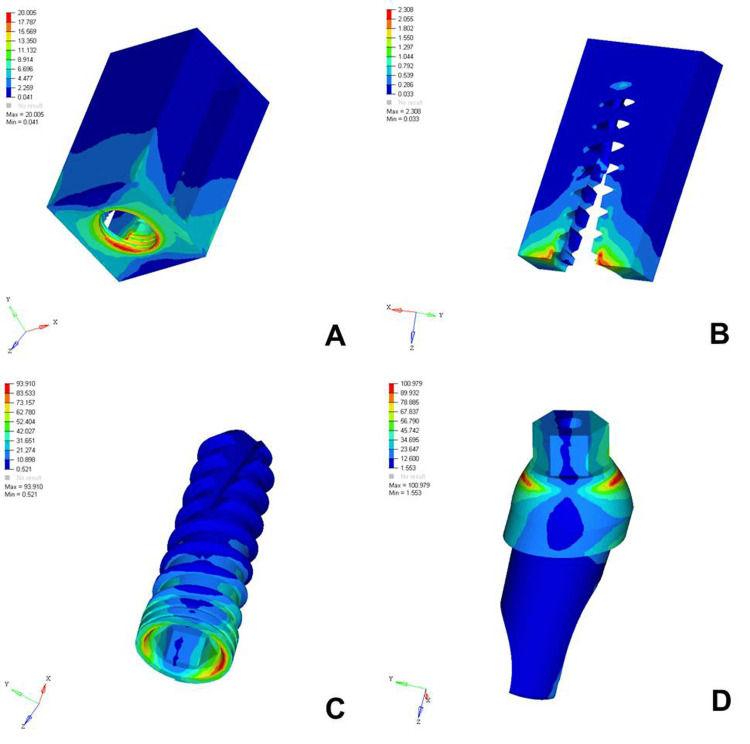
von Mises stresses in megapascals (MPa) in the D1 bone under oblique loading (100 N at 120 degrees) on various structures: A) cortical bone, B) cancellous bone, C) implant, D) abutment. The side bar shows von Mises stress from minimum (blue) to maximum (red) value in MPa. This figure illustrates the finite element analysis (FEA) model developed in the study, taken directly from the ANSYS workbench.

**Figure 4:**
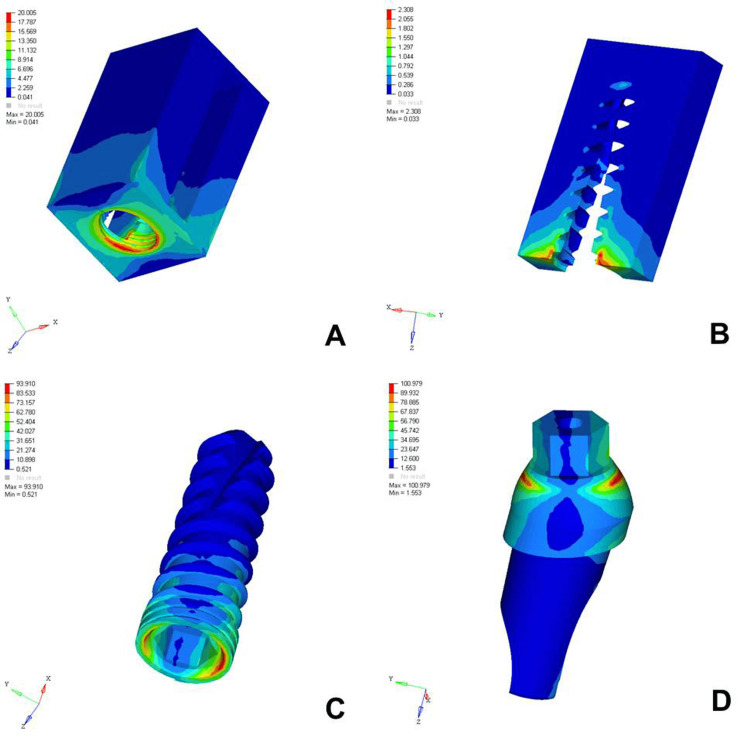
von Mises stresses in megapascals (MPa) in the D2 bone under axial loading (175 N) on various structures: A) cortical bone, B) cancellous bone, C) implant, D) abutment. The side bar shows von Mises stress from minimum (blue) to maximum (red) value in MPa. This figure illustrates the finite element analysis (FEA) model developed in the study, taken directly from the ANSYS workbench.

**Figure 5:**
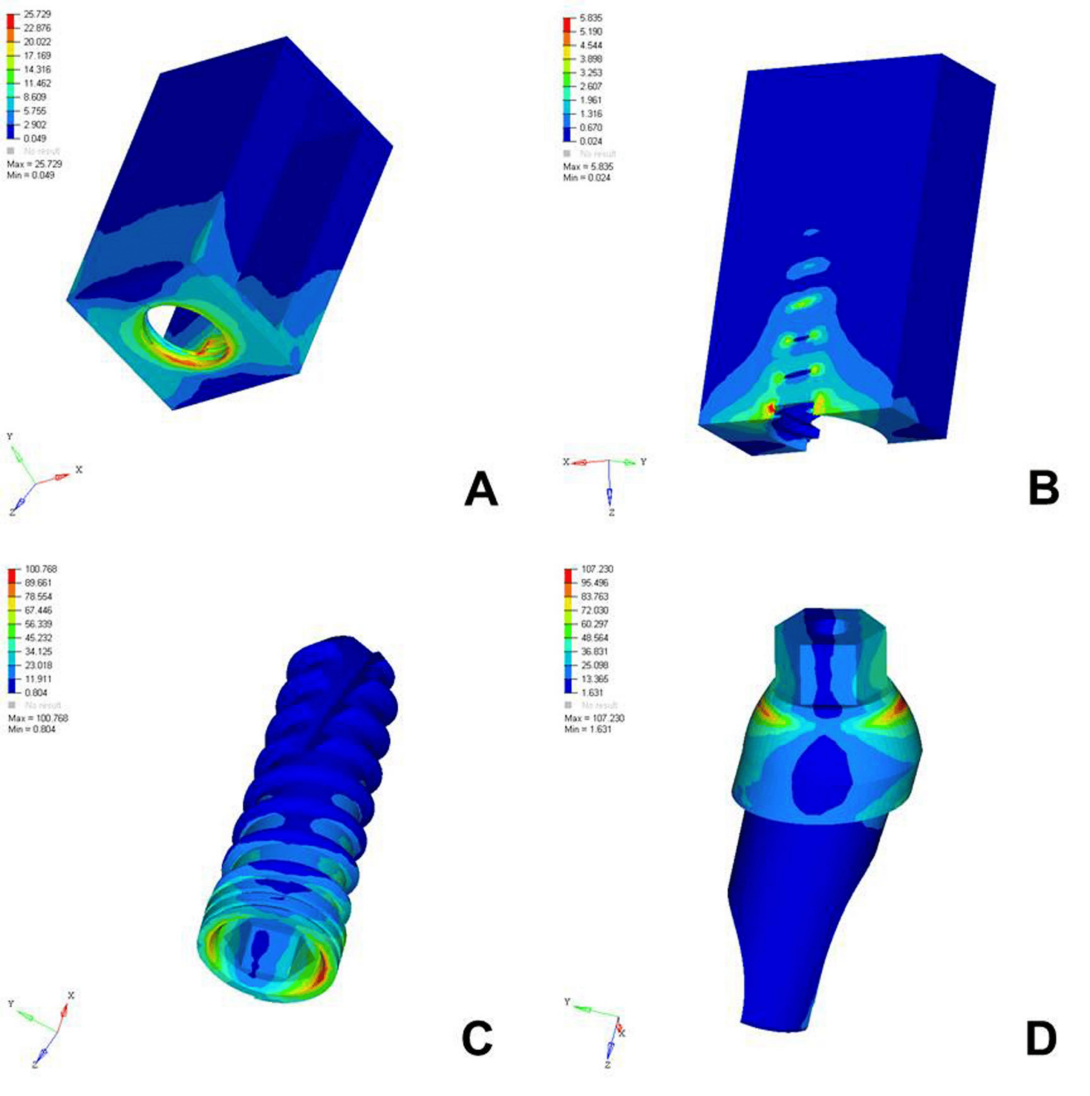
von Mises stresses in megapascals (MPa) in the D2 bone under oblique loading (100 N at 120 degrees) on various structures: A) cortical bone, B) cancellous bone, C) implant, D) abutment. The side bar shows von Mises stress from minimum (blue) to maximum (red) value in MPa. This figure illustrates the finite element analysis (FEA) model developed in the study, taken directly from the ANSYS workbench.

**Figure 6:**
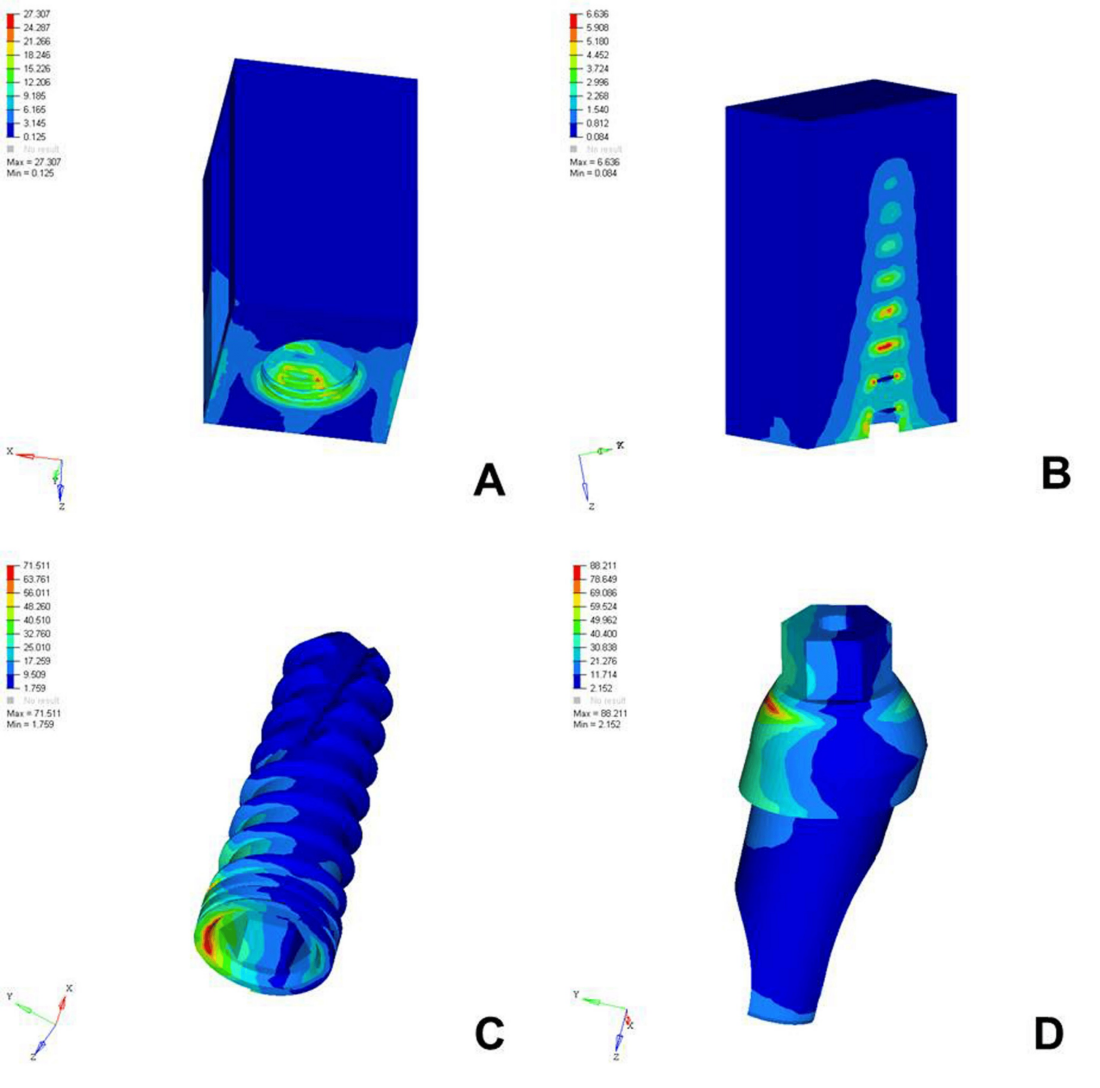
von Mises stresses in megapascals (MPa) in the D3 bone under axial loading (175 N) on various structures: A) cortical bone, B) cancellous bone, C) implant, D) abutment. The side bar shows von Mises stress from minimum (blue) to maximum (red) value in MPa. This figure illustrates the finite element analysis (FEA) model developed in the study, taken directly from the ANSYS workbench.

**Figure 7:**
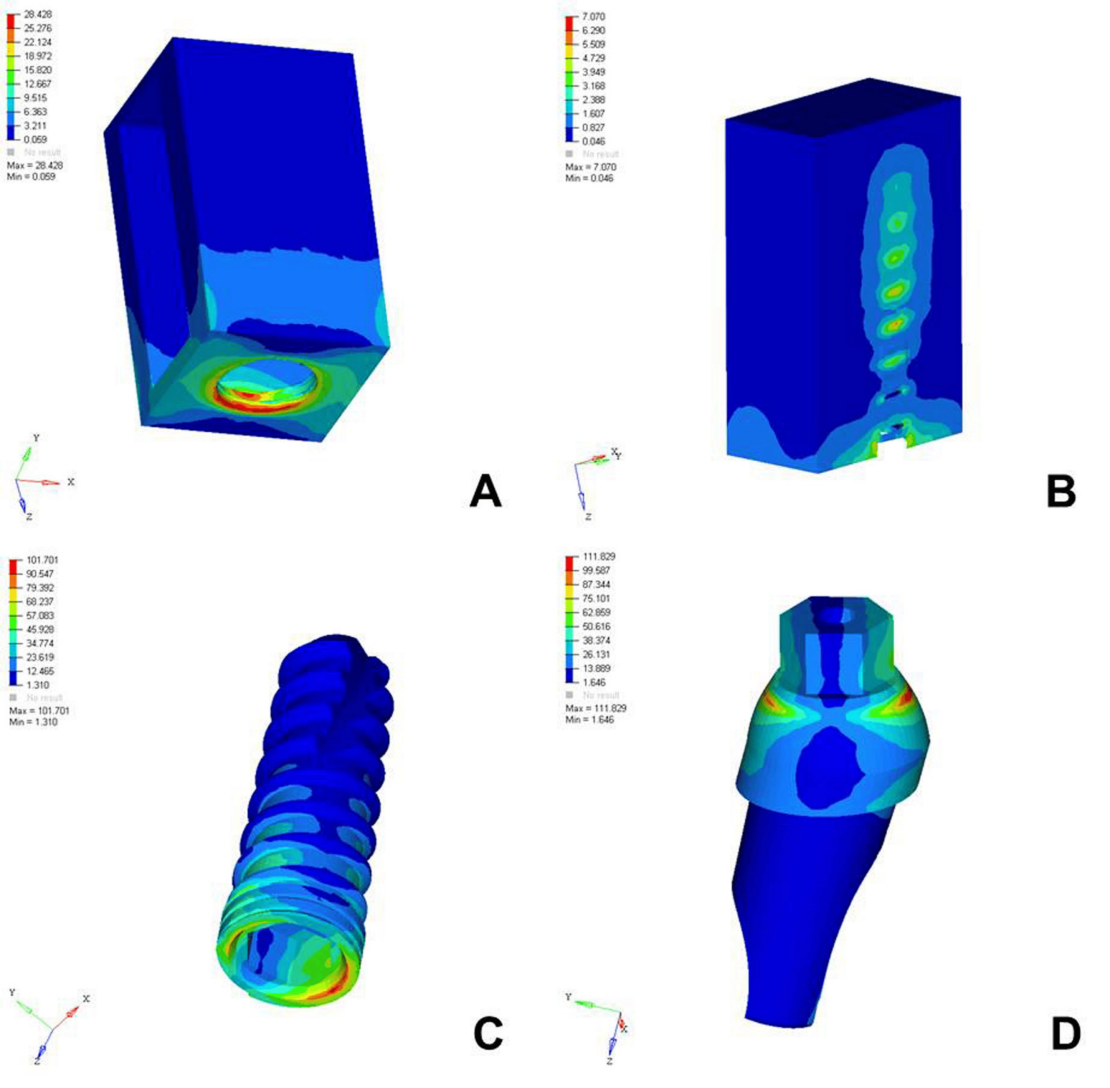
von Mises stresses in megapascals (MPa) in the D3 bone under oblique loading (100 N at 120 degrees) on various structures: A) cortical bone, B) cancellous bone, C) implant, D) abutment. The side bar shows von Mises stress from minimum (blue) to maximum (red) value in MPa. This figure illustrates the finite element analysis (FEA) model developed in the study, taken directly from the ANSYS workbench.

**Figure 8:**
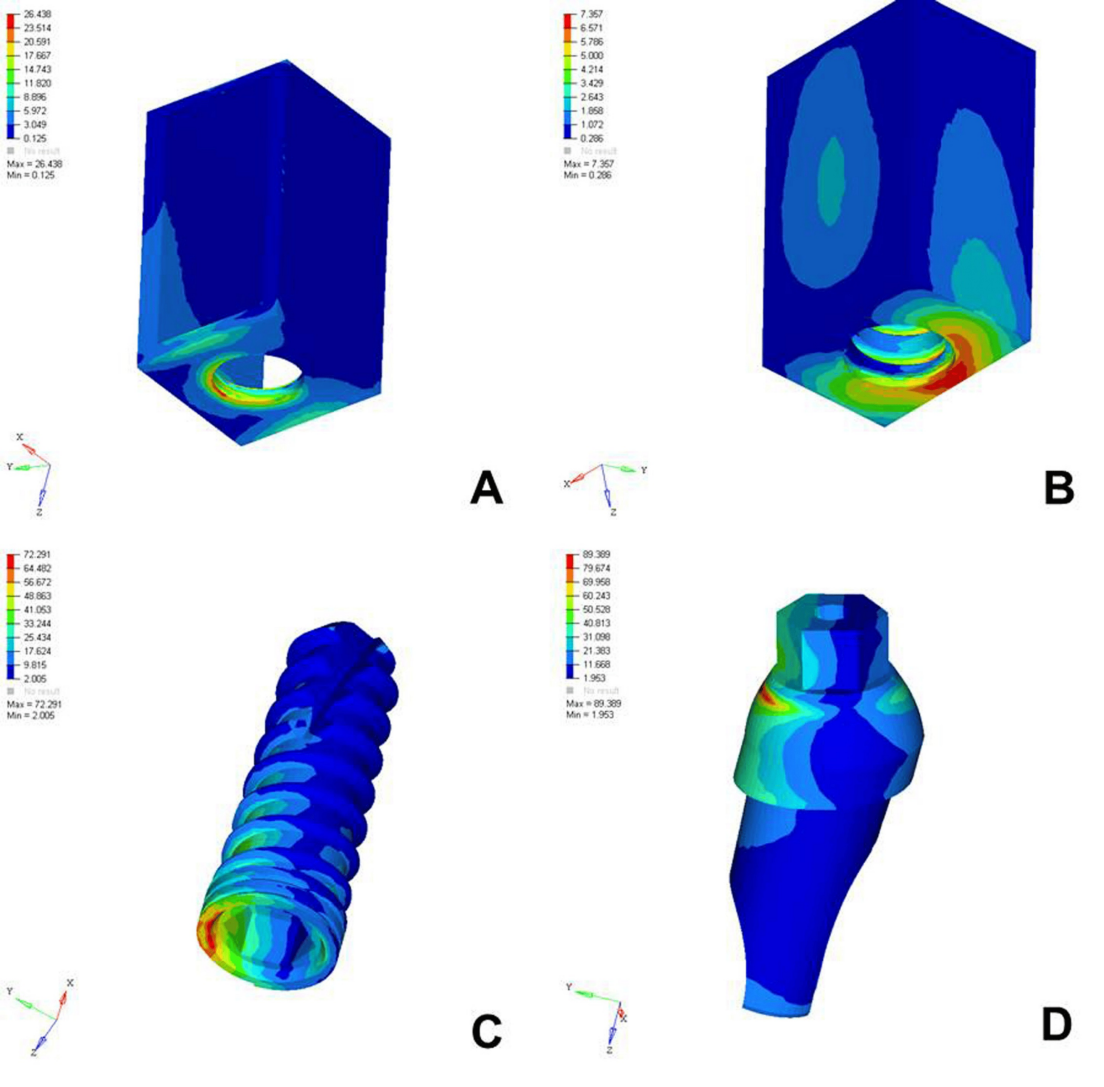
von Mises stresses in megapascals (MPa) in the D4 bone under axial loading (175 N) on various structures: A) cortical bone, B) cancellous bone, C) implant, D) abutment. The side bar shows von Mises stress from minimum (blue) to maximum (red) value in MPa. This figure illustrates the finite element analysis (FEA) model developed in the study, taken directly from the ANSYS workbench.

**Figure 9:**
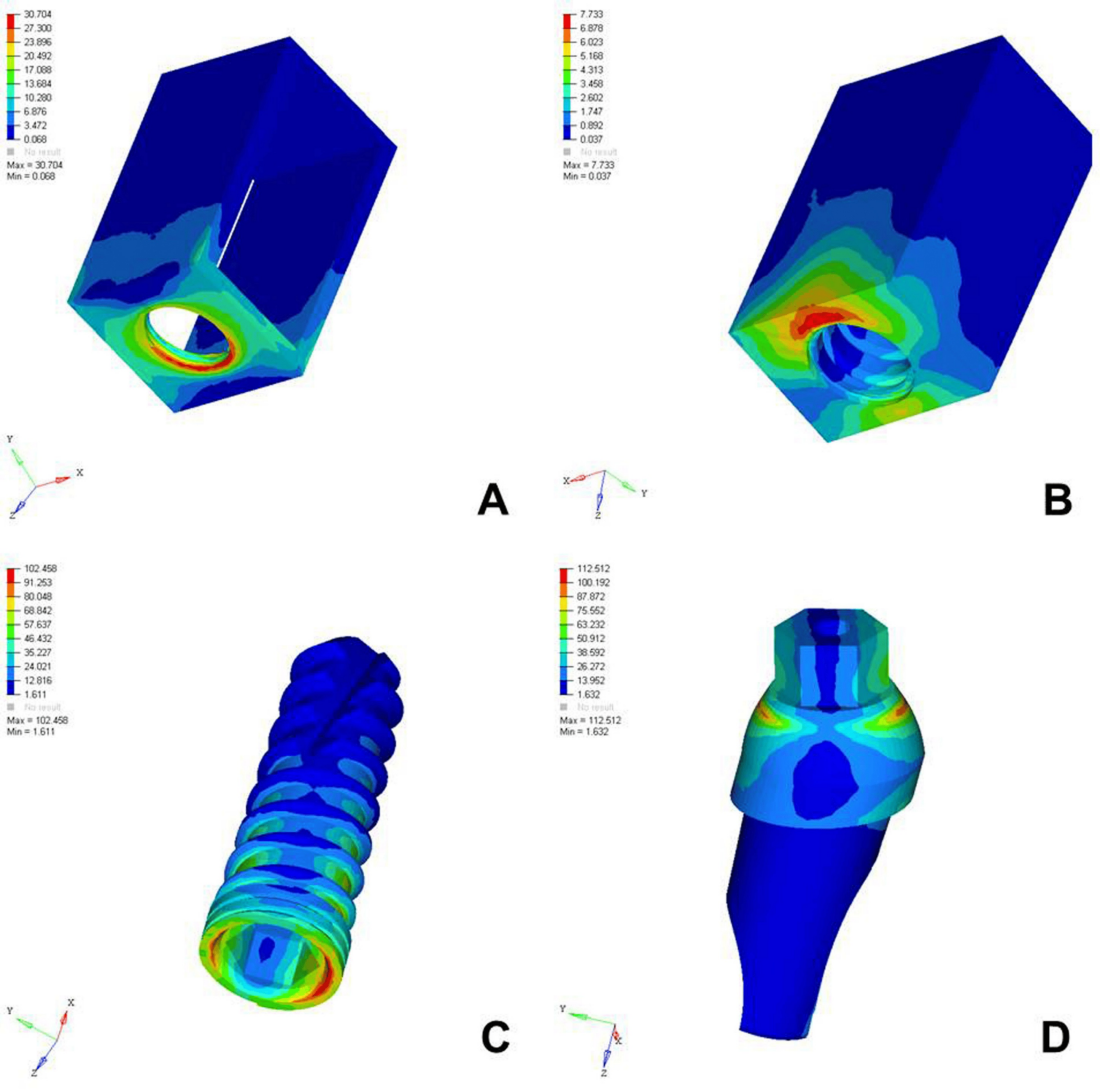
von Mises stresses in megapascals (MPa) in the D4 bone under oblique loading (100 N at 120 degrees) on various structures: A) cortical bone, B) cancellous bone, C) implant, D) abutment. The side bar shows von Mises stress from minimum (blue) to maximum (red) value in MPa. This figure illustrates the finite element analysis (FEA) model developed in the study, taken directly from the ANSYS workbench.

The analysis of von Mises stress distribution under both axial and oblique loading in the D1, D2, D3, and D4 bone models revealed significant differences in stress patterns across the implant components and surrounding bone structures. Under axial loading, the implant displayed concentrated stress at the neck region with a generally uniform distribution, whereas oblique loading resulted in noticeably higher stress values, especially on the side opposite to the direction of force, indicating increased bending moments. The abutment, which already showed elevated stress levels under axial load, particularly at the implant-abutment interface, experienced even more pronounced stress under oblique loading, suggesting a heightened risk of mechanical complications. The abutment screw followed a similar trend, with stress concentrations markedly amplified under oblique forces, reinforcing concerns regarding screw loosening or fracture in nonaxial load scenarios. Stress in the cancellous bone remained low under both conditions but showed a slight increase under oblique loading owing to the lateral component of the force. The cortical bone, which bore significant stress under axial loading, exhibited even higher and more uneven stress concentrations under oblique loading, especially near the crestal region, reflecting its primary role in load transfer and the biomechanical challenges posed by angled forces. Overall, oblique loading introduced greater stress magnitudes and asymmetrical distributions than did axial loading, emphasizing the critical need for careful implant design, angulation, and loading considerations to ensure long-term functional success and stability (Figures 2-9).

The analysis of von Mises stress distribution under axial loading (178 N) across different bone densities (D1-D4) revealed a clear trend in how varying bone quality influenced stress transmission to both implant components and surrounding bone structures. In the cortical bone, the maximum stress increased progressively from D1 (12.023 MPa) to D4 (26.438 MPa), suggesting that as the bone became less dense, the cortical layer was forced to bear a greater load owing to the reduced support from the underlying cancellous bone. Similarly, cancellous bone showed an increase in stress from D1 (1.178 MPa) to D4 (7.357 MPa), reflecting diminished structural integrity and a reduced ability to distribute load effectively in softer bone types. The implant itself experienced a gradual increase in maximum stress from 67.319 MPa in D1 to 72.291 MPa in D4, indicating that a lower bone density led to increased implant loading, possibly because of less efficient stress dissipation. The abutment and abutment screws followed the same pattern, with the abutment showing a slight increase from 86.324 MPa in D1 to 89.389 MPa in D4 and the screw increasing from 20.735 MPa to 23.383 MPa. This overall trend highlighted that as bone density decreased from D1 to D4, all structures, especially the cortical bone and implant components, were subjected to higher stress levels. These findings emphasized the importance of considering bone quality in implant planning, as lower-density bone types might require enhanced implant design, surface treatments, or additional surgical strategies to ensure mechanical stability and long-term success (Table 2).

**Table 2:** von Mises stresses in megapascals (MPa) resulted under axial load (178 N) on the finite element analysis (FEA) models. Max.: maximum von Mises stress, Min.: minimum von Mises stress

Structures	D1 bone	D2 bone	D3 bone	D4 bone
Cortical bone	Max.- 12.023	Max.- 14.914	Max.- 24.341	Max.- 26.438
	Min.- 0.083	Min.- 0.091	Min.- 0.125	Min.- 0.125
Cancellous bone	Max.- 1.178	Max.- 5.354	Max.- 6.636	Max.- 7.357
	Min.- 0.051	Min.- 0.060	Min.- 0.084	Min.- 0.143
Implant	Max.- 67.319	Max.- 70.389	Max.- 71.511	Max.- 72.291
	Min.- 0.991	Min.- 1.347	Min.- 1.759	Min.- 2.005
Abutment	Max.- 86.324	Max.- 87.577	Max.- 88.211	Max.- 89.389
	Min.- 2.076	Min.- 2.010	Min.- 2.094	Min.- 1.812
Abutment Screw	Max.- 20.735	Max.- 21.083	Max.- 21.510	Max.- 23.383
	Min.- 0.083	Min.- 2.676	Min.- 2.618	Min.- 1.887

The analysis of von Mises stress distribution under oblique loading (100 N) across different bone types (D1-D4) revealed a clear pattern of increasing stress as bone density decreased. In the cortical bone, the maximum stress values increased steadily from 20.005 MPa in D1 to 30.704 MPa in D4, indicating that the softer bone forced the denser cortical layer to absorb a greater proportion of the load. Similarly, cancellous bone showed a marked increase in stress from 2.308 MPa in D1 to 7.733 MPa in D4, reflecting the reduced ability of low-density bone to dissipate oblique forces efficiently. The implant experienced a progressive increase in maximum stress from 97.910 MPa in D1 to 102.458 MPa in D4, suggesting that implant structures endured higher loading as the bone quality declined. A similar trend was observed in the abutment, where the stress increased from 100.979 MPa in D1 to 112.512 MPa in D4, and in the abutment screw, which showed a moderate increase from 26.323 to 27.099 MPa. These findings indicated that under oblique loading conditions, the stress magnitudes were amplified in all components as the bone became less dense. This underlines the biomechanical vulnerability of implants in D3 and D4 bones and the necessity for careful load management, prosthetic design, and possible reinforcement techniques to ensure longevity and stability of implant-supported restorations in softer bone types (Table 3).

**Table 3:** von Mises stresses in megapascals (MPa) resulted under oblique load (100 N at 120 degrees) on the finite element analysis (FEA) models. Max.: maximum von Mises stress, Min.: minimum von Mises stress

Structures	D1 bone	D2 bone	D3 bone	D4 bone
Cortical Bone	Max..- 20.005	Max.- 25.729	Max.- 28.428	Max.- 30.704
	Min.- 0.041	Min.- 0.049	Min.- 0.059	Min.- 0.068
Cancellous bone	Max.- 2.308	Max.- 5.835	Max.- 7.070	Max.- 7.733
	Min. -0.033	Min. - 0.024	Min. - 0.031	Min.- 0.020
Implant	Max.- 97.910	Max.- 100.768	Max.- 101.701	Max.- 102.458
	Min.- 0.521	Min.- 0.804	Min.- 1.255	Min.- 1.342
Abutment	Max.- 100.979	Max.- 107.230	Max.- 111.829	Max.- 112.512
	Min.- 1.553	Min.- 1.631	Min.- 1.646	Min.- 1.632
Abutment Screw	Max.- 26.323	Max.- 26.964	Max.- 27.007	Max.- 27.099
	Min.- 2.935	Min.- 3.005	Min.- 3.254	Min.- 3.059

## Discussion

FEA provides valuable insights into the biomechanical behavior of dental implants in the anterior maxilla, particularly under varying bone densities and loading conditions. The results highlight the critical influence of bone quality (D1 to D4) and loading direction (axial versus oblique) on stress and strain distributions, which are pivotal for implant stability and longevity. The results of our study indicated that the maximum stresses were observed at the neck of the implant under axial loading and opposite to the direction of the abutment under oblique loading in all models. Our results for axial loading are in accordance with those of previous studies by Kao et al. [20] and Cardelli et al. [21]. Similarly, the results for oblique loading are in accordance with those of a previous study [22]. Nevertheless, our findings on oblique loading do not align with those of Guven et al. [23] and Bahuguna et al. [24]. In the aforementioned studies, the force was applied directly to the superior aspect of the abutment, whereas in the present investigation, the force was exerted on the palatal surface of the zirconia crown, which accurately reflected the clinical situation. When the orientation of the load is contrary to the orientation of the angled abutment, it results in a reduction in the stress exerted on the adjacent osseous tissue and implant [25]. This outcome indicates that angulated abutments are an appropriate alternative for the rehabilitation of implants positioned at suboptimal sites. Our study further indicated that the stresses were greater on implants than on abutment screws. According to a study by Segundo et al. [26], the internal-hex system led to a higher stress concentration around the implant neck and prevented the accumulation of stress on the abutment screw, as observed in our study.

The observed trend of increasing von Mises stress from D1 to D4 under both axial and oblique loading underscores the significant role of bone density in load dissipation. Cortical bone in the D1 model exhibited the lowest stress (12.023 MPa under axial loading and 20.005 MPa under oblique loading), reflecting its dense structure and superior mechanical support. In contrast, D4 bone, characterized by minimal cortical thickness and soft trabecular structure, showed the highest stress levels (26.438 MPa axially and 30.704 MPa obliquely), indicating a reduced capacity to distribute forces effectively. This is consistent with previous studies, such as those by Sevimay and Turhan [27], who reported higher stress concentrations in low-density bones (D3 and D4) owing to their limited structural integrity. Premnath et al. [16] reported similar results.

The cancellous bone followed a similar pattern, with stress increasing from 1.178 MPa in D1 to 7.733 MPa in D4 under oblique loading. This suggests that in softer bone types, the trabecular component bears a greater proportion of the load, potentially leading to bone resorption or implant micromotion. The implant itself experienced a modest increase in stress from D1 (67.319 MPa axially, 97.910 MPa obliquely) to D4 (72.291 MPa axially, 102.458 MPa obliquely), which may be attributed to reduced bone support and increased bending moments in the softer bone. These findings corroborate the work of Azcarate-Velázquez et al. [28], who noted that implants in D4 bone are more susceptible to mechanical overload and that a decrease in bone quality leads to worse stress distribution and increased stress in the cortical bone, necessitating careful prosthetic planning.

Furthermore, in our study, stress was higher in the cortical bone than in the cancellous bone in all models. This phenomenon can be attributed to variations in the modulus of elasticity between cortical and cancellous bones. Cortical bone, characterized by a higher modulus of elasticity, exhibits greater resistance to deformation and is capable of supporting more load than cancellous bone. Additionally, the elevated stress concentration in the cortical bone can be ascribed to the fact that the mechanical stress distribution predominantly occurs at the bone interface with the implant. The extent of contact between the implant and bone was directly correlated with bone density. Notably, the proportion of bone contact is considerably higher in cortical bone than in cancellous bone [8,10].

Impact of loading conditions

Oblique loading consistently produced higher stress magnitudes and more uneven distributions than axial loading across all the bone types. For instance, cortical bone stress in D4 increased from 26.438 MPa under axial loading to 30.704 MPa under oblique loading, reflecting the biomechanical challenges posed by non-axial forces. This is particularly relevant in the anterior maxilla, where implants are often subjected to angled forces during mastication or speech [6]. The abutment and abutment screws also exhibited amplified stress under oblique loading, with the abutment reaching 112.512 MPa in D4 compared to 89.389 MPa axially. This aligns with a previous systematic review, who highlighted the risk of screw loosening and abutment failure under non-axial forces, particularly in low-density bones [29].

The higher stress under oblique loading can be attributed to increased bending moments and shear forces, which challenge the implant-bone interface and crestal bone integrity [6]. This is especially critical in the D3 and D4 bones, where the thin cortical layer and porous trabecular structure offer limited resistance to lateral forces. These results suggest that clinicians should prioritize strategies to minimize oblique loading in softer bones, such as optimizing abutment angulation or using wider implants to enhance load distribution.

Clinical implications

These findings emphasize the importance of tailoring implant treatment plans to improve the bone quality. In bones D1 and D2, the dense cortical structure provided robust support, allowing for standard implant designs and abutment configurations. However, in D3 and D4 bones, the elevated stress levels necessitate additional considerations, such as surface-modified implants to enhance osseointegration or bone augmentation techniques to improve bone density [2]. The 15^0^ angled abutment used in this study, which is effective for esthetic and anatomical alignment, may exacerbate stress under oblique loading, particularly in D4 bone. Clinicians might consider angulated abutments to reduce stress concentrations, as suggested in previous studies [7].

Moreover, stress concentration in the crestal bone under oblique loading highlights the risk of bone resorption, which is a common cause of implant failure. Techniques such as platform switching or the use of implants with microthreaded necks can mitigate crestal bone stress. For patients with D4 bone, adjunctive procedures such as guided bone regeneration or sinus augmentation may be warranted to enhance the bone quality prior to implant placement [2].

Limitations and future directions

Although this study provided a comprehensive biomechanical analysis, certain limitations must be acknowledged. The assumption of perfect osseointegration and bonded interfaces simplifies the model, but may not fully reflect clinical scenarios where microgaps or incomplete integration occur. In addition, the study focused on static loading conditions, whereas dynamic and cyclic loading were more representative of the masticatory forces. Future studies should incorporate fatigue analysis to assess the long-term implant performance under repeated loading. The material properties were assumed to be isotropic and linear, which may not capture the anisotropic nature of the bone or the viscoelastic behavior of the surrounding tissues. Incorporating more complex material models can enhance the accuracy of stress predictions. Furthermore, the study modeled a single implant, whereas multiple implants or splinted restorations may alter stress distribution. Future research could explore these configurations to provide a more holistic understanding of biomechanical interactions.

## Conclusions

The findings of this study revealed that bone density significantly influenced stress distribution, with softer bone types (D3 and D4) experiencing greater stress concentrations than denser bone types (D1 and D2). Oblique loading amplified stress asymmetrically compared to axial loading, posing a higher risk of mechanical complications, particularly in less dense bones. The angled abutment increased stress at the implant-abutment interface, suggesting the potential for issues such as screw loosening in the softer bone. Clinicians should consider advanced implant designs, surface treatments, or bone augmentation for D3 and D4 bones, along with strategic occlusal planning to minimize oblique forces. These results inform tailored implant placement and prosthetic strategies to enhance biomechanical stability and esthetic outcomes in anterior maxillary implant dentistry. Future studies should explore varied abutment angulations and dynamic loading to further optimize clinical approaches.
